# Is the auditory system cognitively penetrable?

**DOI:** 10.3389/fpsyg.2015.01166

**Published:** 2015-08-11

**Authors:** Berit Brogaard, Dimitria Electra Gatzia

**Affiliations:** ^1^The Brogaard Lab for Multisensory Research, University of MiamiMiami, FL, USA; ^2^Department of Philosophy, University of OsloOslo, Norway; ^3^Department of Philosophy, The University of Akron Wayne CollegeAkron, OH, USA

**Keywords:** auditory perception, cognitive penetration, McGurk illusion, semantic-coherence, top-down influences, top-down modulation, tritone illusion, perceptual learning

According to the hierarchical model of sensory information processing, sensory inputs are transmitted to cortical areas, which are crucial for complex auditory and speech processing, only after being processed in subcortical areas (Hickok and Poeppel, 2007; Rauschecker and Scott, 2009). However, studies using electroencephalography (EEG) indicate that distinguishing simultaneous auditory inputs involves a widely distributed neural network, including the medial temporal lobe, which is essential for declarative memory, and posterior association cortices (Alain et al., 2001; Squire et al., 2004). More recent studies have even demonstrated plasticity of auditory signals as low as the brainstem (Suga, 2008). Collectively, studies suggest that the functional architecture of perceptual processing involves primarily top-down modulation (Suga et al., 2002; Gilbert and Li, 2013; Chandrasekaran et al., 2014). Top-down influences exerted throughout the auditory systems (Lotto and Holt, 2011) include: memory (Goldinger, 1998)^1^, attention (Choi et al., 2014), which has been found to modulate auditory encoding in the cochlea, a subcortical area (Maison et al., 2001), (prior) knowledge of syntax or words (Ganong, 1980; Warren, 1984)^2^, and experience-based expectations pertaining to the speaker's accent (Deutsch, 1996; Deutsch et al., 2004; Irino and Patterson, 2006), gender (Johnson et al., 1999), and vocal folds or tract (Irino and Patterson, 2002; Patterson and Johnsrude, 2008).

While a great deal has been written about the issue of cognitive penetrability in the case of vision, audition has received almost no attention. For example, a corresponding body of evidence for top-down modulation in vision has been used to undermine the Cognitive Impenetrability Thesis (CIT) (see Macpherson, 2012; Siegel, 2012; Wu, 2013; Cecchi, 2014). Brogaard and Gatzia (in press) have argued that top-down modulation on visual processes involving prior-knowledge, experience based expectation, or memory do not threaten the CIT, even after acknowledging that such influences are cognitive in nature (see also Pylyshyn, 1999; Raftopoulos, 2001). The reason is that such top-down influences, although cognitive in nature, are distinct from discursive thoughts that stand in a semantically-coherent relation to the phenomenology or content of experience, for instance, thoughts proceeding by argumentation or reasoning rather than by intuition or implicit hypothesis internal to the visual system^3^. If we insisted that instances of top-down modulation be counted as instances of cognitive penetration, the debate about cognitive penetrability would be trivial and, hence, unmotivated since studies clearly indicate that such top-down modulation in visual (or auditory) perception is extensive. A similar argument can be made in the case of audition.

The CIT has traditionally been understood as a semantic thesis. Accordingly, the information a system computes is not sensitive (in a semantically-coherent way) to one's cognitive states and cannot be altered in a way that bears a logical relation to one's knowledge or reasons (Pylyshyn, 1984, 1999; Raftopoulos, 2009). For example, suppose that you experience a sound as /da-da/ and that causes you to form the belief that the sound is /da-da/. In this case, your belief and your auditory experience are semantically coherent: they have roughly the same content. Suppose now that you acquire the belief that the sound is in fact /ba-ba/ (say, because you have now come to believe that the Cartesian evil genius has made you hear it as /da-da/ when it is in fact a /ba-ba/ sound). According to the semantic thesis, your newly acquired belief, for which you may have ample justification, cannot alter the content computed by your auditory system; you will continue to experience the sound *as* /da-da/ despite that you have come to believe that it is /ba-ba/. Some proponents of the semantic thesis have argued that changes to the information a system computes are attributed to intra-perceptual principles that do not conform to standard tenets of rationality, such as standard rules of logic, probability theory and statistics, or rational choice theory (Brogaard and Gatzia, in press).

Undermining the CIT requires demonstrating that changes in the phenomenology of one's auditory perception are due to the listener's discursive or rational thoughts that stand in the right sort of semantic relation to her experience. So it is not enough that discursive thoughts influence experience; they must do so in a semantically-coherent way. Consider ventriloquism, for example. Suppose that I believe that the puppet is not actually producing the sounds (the person holding the puppet is) but I nevertheless hear the speech as coming from the puppet's mouth. In this case, the content of my belief differs from the content as my auditory experience. Now suppose that my discursive thoughts about what *really* goes on in the case of ventriloquism gives rise to a stress reaction in me (for some reason) and that this mood (the stress) changes the content of my experience: I no longer *hear* the speech as coming from the puppet. In this case, it may appear that my discursive thoughts have changed my auditory experience in a semantically-coherent way: my belief and my experience now have the same content. However, by hypothesis, it is the mood, not my beliefs, that changed my auditory experience. Since moods, unlike beliefs, have no contents, the stress (a mood) cannot have the same content as either my belief or my auditory experience. The content of my experience has thus changed but not in a semantically-coherent way. This semantic-coherence has to be involved in every step of the process for changes in phenomenology to threaten the CIT. For example, if my belief that the puppet is not actually producing the sounds were to cause me to no longer experience the speech as coming from the puppet via a chain of logically related processes, then the content of my belief would have changed the content of my experience in a semantically coherent-way. Such a case would indeed threaten the CIT.

Additionally, cases that involve the *indirect* influencing of auditory experience by beliefs (or discursive thoughts) need not threaten the CIT. For example, Fodor (1988) jokingly said that his heart is cognitively “penetrated” by his intention to do calisthenics since it results in doing calisthenics, resulting in his heart rate increasing. What this joke illustrates is that the locution “receives input from” is not transitive, meaning that it is not the case that if a process B receives input from A, and C received input from B that C receives input from A since it is possible that none of B's outputs that were responses to inputs from A affected C (Lyons, 2015).

Cases of perceptual learning involve such indirect influencing of auditory perception. Typically, perceptual learning refers to the brain's plasticity, i.e., the gradual structural or functional changes in the connectivity of sensory systems resulting from training consisting of repeated exposure to particular stimuli (Roelfsema et al., 2010). However, the competition between verbal and implicit systems (COVIS) model suggests a dual-system framework, according to which learners, in information-integration tasks, initially use the reflective (rule-based) system, but switch to the reflexive (information-integration) system with practice (Maddox et al., 2013; Valentin et al., 2014)^4^. The fact that the reflective system is mediated by the prefrontal cortex and involves hypothesis testing by the learner seems to suggest that at least some cases of perceptual learning may constitute cases of cognitive penetration. This conclusion, however, is too hasty. The reflexive system is viewed as indirect and procedural: trial feedbacks reinforce associations of stimuli located in different regions of perceptual space with specific motor outputs (Maddox et al., 2013). It follows that the changes in auditory phenomenology associated with the reflective system result indirectly from the brain's plasticity, not directly from the listener's discursive thoughts (in a semantically-coherent way). Perceptual learning, therefore, need not threaten the CIT, provided that the changes in phenomenology result indirectly from changes in the brain's plasticity, which cannot be attributed to the listener's discursive thoughts.

Auditory illusions are useful tools to illustrate the inability of our discursive thoughts to alter the phenomenology of our auditory experience in a semantically-coherent way. One example is the tritone illusion. Deutsch (2007) presented listeners with two tones in succession that are opposite in the positions along the pitch class space such as G# followed by D or C followed by F#, which comprised an interval of six semitones (known as tritone). When one of the pairs was played (say, G# followed by D) some of the listeners heard a descending pattern while others heard an ascending pattern. However, when another pair was played (say, C followed by F#) listeners who had previously heard a descending pattern now heard an ascending one and vice versa. The tritone illusion varies in correlation with the accent of the speaker. For example, while Californians tended to hear the pattern as ascending, Britons tended to hear it as descending (Deutsch, 1991). A considerable difference was also observed between mothers who had grown up in widely different geographical regions. Perhaps not surprisingly, significant similarities were observed among these mothers and their children, even though the children had not grown up in the same geographic regions as their mothers (Deutsch, 1996).

The tritone illusion persists even after listeners are informed that the two tones in succession are opposite in the positions along the pitch class space, indicating that their discursive thoughts cannot alter the phenomenology of their auditory experiences. What one hears depends on the configuration of one's auditory system, which is, among other things, subject to developmental influences (Deutsch et al., 2004). However, top-down modulation caused by adaptation- or development-based knowledge, experience-based expectation, memory, or attention are consistent with the claim that auditory perception is not cognitively penetrable, at least not in any interesting sense, as the changes in phenomenology cannot plausibly be attributed to the listener's discursive thoughts.

Another example is the McGurk illusion, which arises when auditory speech cues are presented in synchrony with incongruent visual speech cues (McGurk and MacDonald, 1976). For example, when the auditory syllable “ba” is presented in synchrony with a speaker mouthing “ga,” subjects typically report hearing “da.” However, when the auditory syllable “ga” is presented in synchrony with a speaker mouthing “ba,” subjects typically report hearing “bga”^5^. As with the tritone illusion, the McGurk illusion persists even after subjects are informed that the auditory syllable is “ba” in the first case and “ga” in the second. Windmann (2004) found that the clarity and, to some extent, the probability of the illusion was significantly influenced by the listener's experience-based expectations, which do not threaten the CIT for the same reason: the information the system computes is not altered by the listener's discursive thoughts.

It may nevertheless be objected that other cases such as sine wave speech appear to threaten the CIT since they seem to involve changes in phenomenology which can be attributed to subject's discursive thoughts^6^. For example, naive listeners tend to hear sine wave speech as tones or whistles, rather than speech. After being familiarized with the linguistic message, however, many listeners readily hear sine wave as speech (Sheffert et al., 2002). However, it is not clear, in this case, whether it is the listener's beliefs that cause a change in her experience. For example, it could be that such cases involve cognitive penetration if the listener's belief about the content of the linguistic message were to alter (in a semantically-coherent way) the phenomenology of the listener's experience. Or, it could be that the listener is still hearing the same tones or whistles but interprets them on the basis of the newly acquired knowledge of the linguistic message. The more likely explanation is that it is a case of normalization based on experience-based expectation given that the listener comes to understand sine wave speech only *after* learning its linguistic message. So it seems that the expectation that the sound has the linguistic message the listener expects it to have is what is doing all the work. Indeed, studies suggest that listeners use a range of information regarding the speaker, including the speaker's supposed nationality (Niedzielski, 1999), to create a frame of reference to be used during perception in order to normalize what is heard. In other words, listeners utilize adaptation- or development-based knowledge, experience-based expectation, memory, or attention to make sense of speech. However, as we have argued, such changes in phenomenology cannot plausibly be attributed to the listeners' discursive thoughts (at least not in a semantically-coherent way) and, thus, do not threaten the CIT.

## Conflict of interest statement

The authors declare that the research was conducted in the absence of any commercial or financial relationships that could be construed as a potential conflict of interest.

## Footnotes

^1^It has been suggested that the mechanism underlying auditory restoration (the auditory system's ability to compensate for expected missing sounds, see Warren, 1984) involves episodic memory, which involves memory traces left by an experience that are activated, according to the similarity with the stimulus, when a new stimulus such as a word is heard (see Goldinger, 1998).

^2^As the Ganong effect illustrates, phonemes such as /t/ or /d/ tend to be heard as /t/ when followed by “ask” to form “task” but as /d/ when followed by “usk” to form “dusk.”

^3^Constancy computations, for example, are not obligatorily linked to experiencing sensibles and may precede it (Kentridge et al., 2014).

^4^We thank an anonymous reviewer for helpful comments on the issue of perceptual learning.

^5^Here too it is due to the non-transitivity of the locution “receives input from” that we cannot say that auditory processing is cognitively penetrated by visual processing (see Lyons, 2015).

^6^We thank an anonymous reviewer for posing this question.
